# *Schistosoma mansoni* Vector Snails in Antigua and Montserrat, with Snail-Related Considerations Pertinent to a Declaration of Elimination of Human Schistosomiasis

**DOI:** 10.4269/ajtmh.20-0588

**Published:** 2020-09-08

**Authors:** Martina R. Laidemitt, Sarah K. Buddenborg, Lowell L. Lewis, Lionel E. Michael, Maria J. Sanchez, Reynold Hewitt, Eric S. Loker

**Affiliations:** 1 Center for Evolutionary and Theoretical Immunology, Parasite Division, Museum of Southwestern Biology, Department of Biology, University of New Mexico, Albuquerque, New Mexico;; 2 Wellcome Sanger Institute, Cambridgeshire, United Kingdom;; 3 Government of Montserrat, Brades, Montserrat;; 4 Environmental Health Division, Ministry of Health and Social Development, Road Town, Tortola, British Virgin Islands;; 5 Pan American Health Organization, Washington, District of Columbia;; 6 One Health Center for Zoonoses and Tropical Veterinary Medicine, Ross University School of Veterinary Medicine, Basseterre, Saint Kitts and Nevis

## Abstract

Investigations leading to a WHO-validated declaration of elimination of schistosomiasis transmission are contemplated for several countries, including Caribbean island nations. With assistance from the Pan American Health Organization, we undertook freshwater snail surveys in two such nations, Antigua and Barbuda, and Montserrat in September and October 2017. Historically, the transmission of *Schistosoma mansoni* supported by the Neotropical vector snail *Biomphalaria glabrata* occurred in both countries. Transmission on the islands is thought to have been interrupted by the treatment of infected people, improved sanitation, introduction of competitor snails, and on Montserrat with the eruption of the Soufrière volcano which decimated known *B. glabrata* habitats. Guided by the available literature and local expertise, we found *Biomphalaria* snails in seven of 15 and one of 14 localities on Antigua and Montserrat, respectively, most of which were identified anatomically and molecularly as *Biomphalaria kuhniana*. Two localities on Antigua harbored *B. glabrata*, but no schistosome infections in snails were found. For snail-related aspects of validation of elimination, there are needs to undertake basic local training in medical malacology, be guided by historical literature and recent human schistosomiasis surveys, improve and validate sampling protocols for aquatic habitats, enlist local expertise to efficiently find potential transmission sites, use both anatomical and molecular identifications of schistosomes or putative vector snail species found, if possible determine the susceptibility of recovered *Biomphalaria* spp. to *S. mansoni*, publish survey results, and provide museum vouchers of collected snails and parasites as part of the historical record.

## INTRODUCTION

In 2017, the World Health Assembly adopted WHA70.16, which proposed reinforcing snail control as part of the strategy to eliminate human schistosomiasis as a public health problem.^1^ This is a daunting task because as estimated by the WHO, schistosomiasis transmission occurs in 78 countries, and at least 229 million people required preventive treatment in 2018.^1^ An estimated 1.6 million individuals with intestinal schistosomiasis caused by *Schistosoma mansoni* reside in the Americas.^1^ Although schistosomiasis transmission persists in Brazil, Suriname, and Venezuela,^1,2^ its current status on several Caribbean islands formerly known to be endemic is currently not well understood.^3,4^ Some nations in the Caribbean are considered to have interrupted *S. mansoni* transmission and have been taken off the list of countries with endemic transmission, including Antigua and Barbuda, the Dominican Republic, Guadeloupe, Martinique, Puerto Rico, and Montserrat.^3,5^ However, these claims require further verification, including whether the major *S. mansoni* snail vector in the region, *Biomphalaria glabrata*, is still present. Although schistosomiasis can surely be eliminated in locations where vector snail species persist, such as in Japan, continuing presence or not of susceptible vector species in a country targeted for elimination obviously remains a pertinent consideration. *Biomphalaria glabrata* was once known from at least 12 Caribbean islands, but its continuing presence on most has been questioned.^6,7^ Ecological changes, particularly the introduction of exotic competitor freshwater snails such as *Melanoides tuberculata* and *Thiara granifera*, are believed to be responsible for sharp declines in *B. glabrata* abundance.^7^ Additional species of *Biomphalaria* are also known from the Caribbean islands,^6,8^ and their status as possible hosts for *S. mansoni* there requires further study.

As part of an early stage in the consideration of declaration of elimination of schistosomiasis transmission in the Caribbean, the Pan American Health Organization (PAHO) supported an exploratory survey of two islands, Antigua and Montserrat, with respect to the status of schistosome vector snails currently present. Barbuda was not sampled as Hurricane Irma decimated much of the island in September and October 2017. *Schistosoma mansoni* was first established in Antigua and Barbuda and Montserrat following the influx of people of African descent into the Neotropics in the fifteenth century and was efficiently transmitted by indigenous populations of *B. glabrata.*^9,10^ Studies from Antigua in the 1920s found approximately 18% of people were infected with *S. mansoni* in St. John Parish and a 1930s survey found 60% of people living in St. John Parish near Bendel’s stream and Body Pond were infected.^4,9,11^ These locations were sites where *B. glabrata* was also found.^9^ The prevalence of *S. mansoni* in Antigua then declined over the course of the twentieth century. By the 1980s, the prevalence of *S. mansoni* declined to less than 1%, likely in response to the introduction of *T. granifera* and infrastructure improvements.^9,12,13^

Surveys conducted in the late 1970s and 1980s on Montserrat found 10–14% of people were infected with *S. mansoni* in villages on the island’s windward eastern side^10,14,15^ where *B. glabrata* habitats were also found.^9^ These villages were Trants, Farms, Bethel, Bramble, and Tuitts.^4,14^ In 1995, the Soufrière Hills volcano that dominates the island’s landscape erupted and left the southern half of the island devastated.^16^ The habitats where *B. glabrata* were present on the eastern slopes of the volcano were likely destroyed by pyroclastic flows. The eruption of the volcano, in combination with the introduction of competitor snails such as *M. tuberculata*, is believed to have eliminated *B. glabrata* from the island.^15,17^ Although both Antigua and Montserrat are considered to no longer have endemic transmission of *S. mansoni*, parasitological confirmation is lacking. In addition, there is an absence of recent published surveys of freshwater snails in either country.

Eventual confirmation of the elimination of human schistosomiasis in the Caribbean is of importance in several regards. It would mark the end of an underappreciated health threat to local populations, especially children. It would also preclude infection risks for the millions of people who visit the Caribbean each year. Recent experiences in Corsica show how tourist populations can be at risk of schistosomiasis^18^ and highlight the need for surveillance. Also, efforts on the islands of the Caribbean can help to establish protocols and procedures that might prove valuable when elimination and subsequent validation efforts are eventually undertaken in mainland countries.

In September and October 2017, we visited Antigua and Montserrat with the aim of first providing workers from the respective ministries of health with basic malacology training, and then conducting snail surveys looking for living *Biomphalaria* snails in selected localities. The basic training was designed to encourage and facilitate future additional searches. In this study, the surveys are reported and observations and future strategies and needs relevant to malacological aspects of the eventual declaration of elimination of schistosomiasis are outlined. The focus of this article was on snail-related aspects of validation of the elimination process, with the realization that separate efforts to document infection or transmission in resident human, or domestic or wild mammal, populations will require expertise from different teams of investigators.

## MATERIALS AND METHODS

### Sampling.

**Figure 1.**
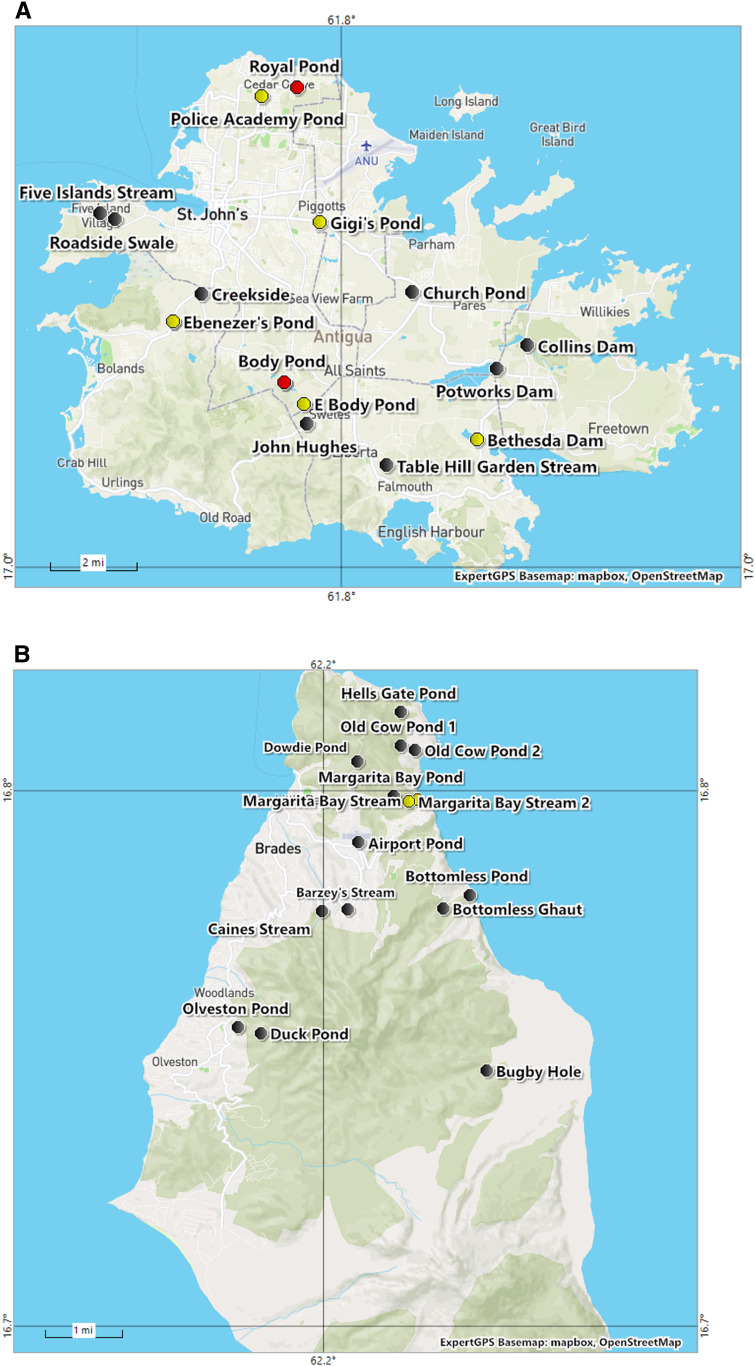
ExpertGPS basemaps of Antigua (**A**) and Montserrat (**B**), and the localities we sampled for aquatic snails. Highlighted with a yellow circle are localities where *Biomphalaria kuhniana* were collected, and highlighted in a red circle are localities where *Biomphalaria glabrata* were collected. Localities with black circles are non-*Biomphalaria* sites. This figure appears in color at www.ajtmh.org.

We collected snails from 15 different freshwater localities in Antigua and 14 localities from Montserrat between September and October 2017 (Table 1). We did not visit Barbuda because Hurricane Irma had recently inflicted heavy damage, causing most people on the island to evacuate. At each locality, we recorded GPS coordinates, habitat type, altitude, distinctive features, and the presence of people and/or animals, and collected freshwater snails (Figure 1). Aquatic snails were collected along the water’s edge using kitchen sieves to sweep aquatic vegetation or a long-handled metal net to scoop along the substrate, rocks, and aquatic vegetation in deeper water. Snails were also picked off submerged rocks, plants, sticks, or debris using forceps. Collection at each locality lasted between 30 minutes and 1 hour. The time spent at each site varied because of the size of the habitat and to ensure thorough collection measures were completed. After collection, debris was removed from snail shells with a Kimwipe™ (Kimberly-Clark Corp., Irvine, TX) and were rinsed with clean water. Snails were placed individually into 12-well tissue culture plates in 3 mL of water. The tissue culture plates were placed in ambient light and left overnight to induce shedding of cercariae as some cercariae may shed at night. The plates were then screened for cercariae using a dissecting scope. Keys were used for the identification of snails^6^ and their trematodes.^19^ Snails and cercariae were fixed in 95% ethanol for later molecular analysis. Some snails were relaxed using menthol crystals and, following the procedures of Pan,^20^ were removed from their shells and fixed in Railliet–Henry solution to facilitate dissections and anatomical observations.

**Table 1:** Malacological collection information from Antigua and Montserrat (vouchers were deposited in the Museum of Southwestern Biology)

Snail Species	Number of Snails	Trematode Infection	Locality	Nation	Latitude	Longitude	Museum Voucher
Ancylidae	3	None shedding	Ebenezer's Pond	Antigua and Barbuda	17.082383	-61.858617	MSB:Host:22426
Ancylidae	1	None shedding	Royal Pond	Antigua and Barbuda	17.062083	-61.819883	MSB:Host:22371
*Biomphalaria glabrata*	1	None shedding	Body Pond	Antigua and Barbuda	17.062083	-61.819883	MSB:Host:22414
*Biomphalaria glabrata*	1	None shedding	Royal Pond	Antigua and Barbuda	17.062083	-61.819883	MSB:Host:22388
*Biomphalaria kuhniana*	62	None shedding	Bethesda Dam	Antigua and Barbuda	17.042867	-61.752583	MSB:Host:22382
*Biomphalaria kuhniana*	46	1 Echinostome and 1 Strigeid	Body Pond	Antigua and Barbuda	17.062083	-61.819883	MSB:Host:22377
*Biomphalaria kuhniana*	49	None shedding	Ebenezer's Pond	Antigua and Barbuda	17.082383	-61.858617	MSB:Host:22381
*Biomphalaria kuhniana*	3	None shedding	Gigi's Pond	Antigua and Barbuda	17.115783	-61.807317	MSB:Host:22379
*Biomphalaria kuhniana*	107	2 Strigeids	Police Academy Pond	Antigua and Barbuda	17.1581	-61.827567	MSB:Host:22384
*Drepanotrema depressissium*	5	None shedding	Body Pond	Antigua and Barbuda	17.062083	-61.819883	MSB:Host:22409
*Drepanotrema depressissium*	8	None shedding	E Body Pond	Antigua and Barbuda	17.054817	-61.812933	MSB:Host:22410
*Drepanotrema surinamense*	9	None shedding	Ebenezer's Pond	Antigua and Barbuda	17.082383	-61.858617	MSB:Host:22422
*Drepanotrema surinamense*	11	None shedding	Police Academy Pond	Antigua and Barbuda	17.1581	-61.827567	MSB:Host:22372
*Gundlachia*	1	1 Strigeid	Body Pond	Antigua and Barbuda	17.062083	-61.819883	MSB:Host:22407
Hydrobiid	1	None shedding	Collins Dam	Antigua and Barbuda	17.074317	-61.73495	MSB:Host:22416
*Melanoides tuberculata*	20	None shedding	Creekside	Antigua and Barbuda	17.091783	-61.8487	MSB:Host:22419
*Melanoides tuberculata*	100	None shedding	E Body Pond	Antigua and Barbuda	17.054817	-61.812933	MSB:Host:22411
*Melanoides tuberculata*	25	None shedding	Ebenezer's Pond	Antigua and Barbuda	17.082383	-61.858617	MSB:Host:22424
*Melanoides tuberculata*	9	None shedding	Five Islands Stream	Antigua and Barbuda	17.118767	-61.883917	No voucher
*Melanoides tuberculata*	30	None shedding	John Hughes	Antigua and Barbuda	17.04805	-61.811917	No voucher
*Melanoides tuberculata*	Several	None shedding	Roadside Swale	Antigua and Barbuda	17.1166	-61.878833	No voucher
No snails found	-	-	Church Pond	Antigua and Barbuda	17.0923	-61.774967	-
No snails found	-	-	Potworks Dam	Antigua and Barbuda	17.066667	-61.745383	-
No snails found	-	-	Table Hill Garden Stream	Antigua and Barbuda	17.034017	-61.784133	-
*Physa acuta*	10	None shedding	E Body Pond	Antigua and Barbuda	17.054817	-61.812933	MSB:Host:22404
*Physa marmorata*	29	1 Strigeid	Body Pond	Antigua and Barbuda	17.062083	-61.819883	MSB:Host:22397
*Physa marmorata*	2	None shedding	Collins Dam	Antigua and Barbuda	17.074317	-61.73495	MSB:Host:22415
*Physa marmorata*	2	None shedding	Creekside	Antigua and Barbuda	17.091783	-61.8487	MSB:Host:22420
*Physa marmorata*	34	None shedding	Ebenezer's Pond	Antigua and Barbuda	17.082383	-61.858617	MSB:Host:22425
*Physa marmorata*	60	None shedding	Five Islands Stream	Antigua and Barbuda	17.118767	-61.883917	MSB:Host:22430
*Physa marmorata*	12	None shedding	Gigi's Pond	Antigua and Barbuda	17.115783	-61.807317	MSB:Host:22432
*Physa marmorata*	3	None shedding	John Hughes	Antigua and Barbuda	17.04805	-61.811917	No voucher
*Physa marmorata*	93	1 Xiphidiocercariae	Police Academy Pond	Antigua and Barbuda	17.1581	-61.827567	MSB:Host:22374
*Physa marmorata*	Several	None shedding	Roadside Swale	Antigua and Barbuda	17.1166	-61.878833	No voucher
*Physa marmorata*	82	None shedding	Royal Pond	Antigua and Barbuda	17.062083	-61.819883	MSB:Host:22373
*Planorbella duryi*	127	None shedding	Collins Dam	Antigua and Barbuda	17.074317	-61.73495	MSB:Host:22386
*Pseudosuccinea columella*	13	None shedding	Body Pond	Antigua and Barbuda	17.062083	-61.819883	MSB:Host:22398
*Pseudosuccinea columella*	2	None shedding	Ebenezer's Pond	Antigua and Barbuda	17.082383	-61.858617	MSB:Host:22428
*Pseudosuccinea columella*	1	None shedding	Gigi's Pond	Antigua and Barbuda	17.115783	-61.807317	MSB:Host:22431
*Pseudosuccinea columella*	9	None shedding	Royal Pond	Antigua and Barbuda	17.062083	-61.819883	MSB:Host:22370
*Biomphalaria kuhniana*	49	None shedding	Margarita Stream	Montserrat	16.7982	-62.181583	MSB:Host:22390
*Drepanotrema sp*	40	None Shedding	Dodie Pond	Montserrat	16.805317	-62.19335	MSB:Host:22421
*Melanoides tuberculata*	30	None shedding	Barzey's Stream	Montserrat	16.777733	-62.195217	No voucher
*Melanoides tuberculata*	Several	None shedding	Bottomless Ghaut	Montserrat	16.777967	-62.176733	No voucher
*Melanoides tuberculata*	6	None shedding	Bottomless Pond	Montserrat	16.777967	-62.171583	No voucher
*Melanoides tuberculata*	8	None shedding	Bugby Hole	Montserrat	16.777967	-62.16825	No voucher
*Melanoides tuberculata*	Several	None shedding	Cane's Ghaut	Montserrat	16.777967	-62.20025	No voucher
*Melanoides tuberculata*	3	None shedding	Dodie Pond	Montserrat	16.777967	-62.19335	No voucher
*Melanoides tuberculata*	13	None shedding	Margarita Stream	Montserrat	16.79895	-62.18625	MSB:Host:22393
*Melanoides tuberculata*	2	None shedding	Olveston Pond	Montserrat	16.75575	-62.2165	MSB:Host:22402
*Physa marmorata*	88	Echinostomes and Strigeids	Airport Pond	Montserrat	16.790233	-62.193083	MSB:Host:22396
*Physa marmorata*	10	None Shedding	Duck Pond	Montserrat	16.754667	-62.212083	MSB:Host:22395
*Physa marmorata*	180	None shedding	Margarita Pond	Montserrat	16.79895	-62.18625	MSB:Host:22435
*Physa marmorata*	4	None shedding	Margarita Stream	Montserrat	16.79895	-62.18625	MSB:Host:22394
*Physa marmorata*	20	None shedding	Old Cow Pond	Montserrat	16.808383	-62.184867	MSB:Host:22437
*Physa marmorata*	3	None shedding	Olveston Pond	Montserrat	16.75575	-62.2165	MSB:Host:22368
*Physa sp*	1	None shedding	Barzey's Stream	Montserrat	16.777733	-62.195217	No voucher

*B. glabrata* = *Biomphalaria glabrata*; *B. kuhniana* = *Biomphalaria kuhniana*; *D. depressissium* = *Drepanotrema depressissium*; *D. surinamense* = *Drepanotrema surinamense*; *M. tuberculata* = *Melanoides tuberculata*; MSB = The Museum of Southwestern Biology; *P. columella* = *Pseudosuccinea columella*; *P. marmorata* = *Physa marmorata*.

### Molecular characterization of *Biomphalaria* and closely related snails.

**Figure 2.**
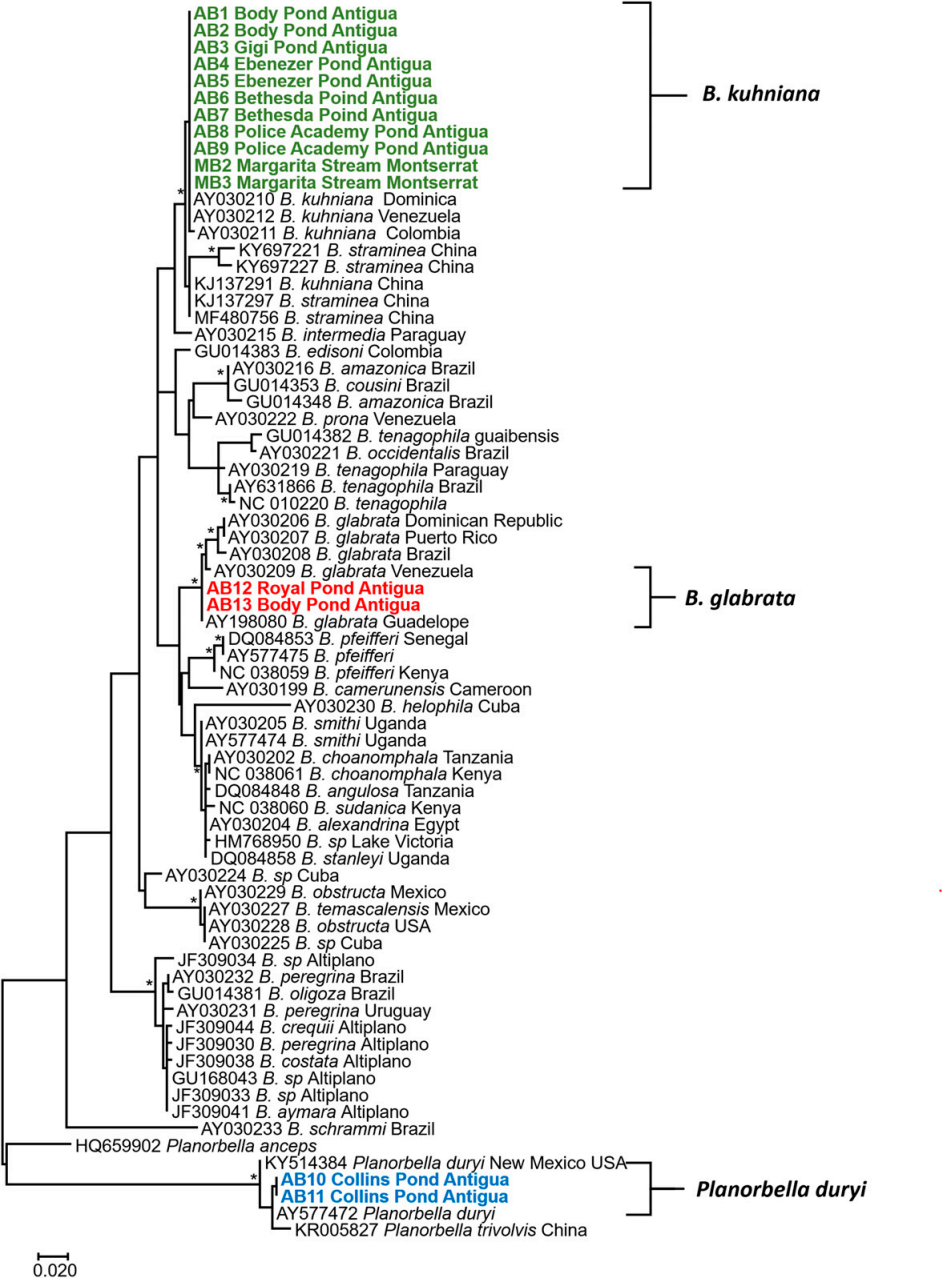
Phylogenetic tree based on 461 positions of the 16S rDNA gene. A total of 1,000 bootstraps were run, and model GTR + I + G was selected via model selection. * denotes nodes greater than 90%. Specimens obtained from Antigua and Montserrat are shown in bold and colored according to different species. This figure appears in color at www.ajtmh.org.

Partial sequences of the 16S rRNA and internal transcribed spacers one and two (ITS1 and ITS2) were amplified by PCR. Snail genomic DNA was extracted from one or two specimens from each locality (16 specimens in total) using the ENZA Mollusc Kit (Omega Bio-Tek, Norcross, CA). The primers used in this study were used to compare and differentiate *Biomphalaria* sequences obtained from GenBank and collected in this study (Figures 1 and 2). The internal transcribed spacer was amplified using ITS1-S, 5′ CCATGAACGAGGAATTCCCAG 3′; BD2, 5′ TATGCTTAAATTCAGCGGGT 3′; and ITS2.2, 5′ CCTGGTTAGTTTCTTTTCCTCCGC3′ primers.^21^ The 16S region was amplified using 16Sar, 5′ CGCCTGTTTATCAAAAACAT 3′ and 16Sbr, 5′ CCGGTCTGAACTCAGATCACGT3′ primers.^22^ The volume of each PCR was 25 µL, with 1 µL of 100 ng of DNA, 0.8 mM/L of deoxynucleotides, 2.5 mM/L of MgCl_2_, 0.2 units of Ex Taq DNA (Clontech, Mountain View, CA), and 0.4 µM/L of each primer. PCR cycles for both genes followed by study by Palumbi.^22^

PCR fragments were separated by agarose gel electrophoresis and visualized with 0.5% GelRed™ nucleic acid gel stain (Biotium Inc., Hayward, CA) and were purified using the ExoSap-IT (Applied Biosystems, Foster City, CA). Both strands were sequenced using an Applied Biosystems 3130 automated sequencer and BigDye Terminator Cycle Sequencing Kit version 3.1 (Applied Biosystems). DNA sequences were verified by aligning reads from the 5′ and 3′ directions using Sequencher 5.0 and manually corrected for ambiguous base calls (Gene Codes, Ann Arbor, MI). Approximately 1,149 bases were generated from ITSs and 470 bases from the 16S gene. Sequences were aligned using CLUSTAL W, and the best fit model of substitution for both genes was modeled in Molecular Evolutionary Genetics Analysis7.^23^ Phylogenetic analyses using maximum likelihood (ML) included our 13 samples along with 43 sequences from National Center for Biotechnology Information-GenBank for ITSs and 60 for 16S. A total of 1,277 positions were used for ITS alignment and 413 positions for the 16S alignments. Heuristic searchers were used for ML analyses, and 1,000 bootstrap replicates were run for each dataset. Sequences generated in this study were submitted to GenBank. Our specimens were also deposited as vouchers in the Museum of Southwestern Biology (Table 1).

Molecular detection for prepatent (not shedding) *S. mansoni* infections from the previously extracted *Biomphalaria* was also performed. We tested if we could amplify *S. mansoni* or *Schistosoma rodhaini* DNA using the nicotinamide adenine dinucleotide dehydrogenase subunit 5 (*ND*5) PCR assay described by Lu et al.^24^ This is a sensitive assay (> 0.1 fg DNA) and differentiates *Schistosoma* species either by band size or absence/presence. We followed the same PCR gel detection protocol as described by Lu et al.^24^

## RESULTS

### Antigua.

**Figure 3.**
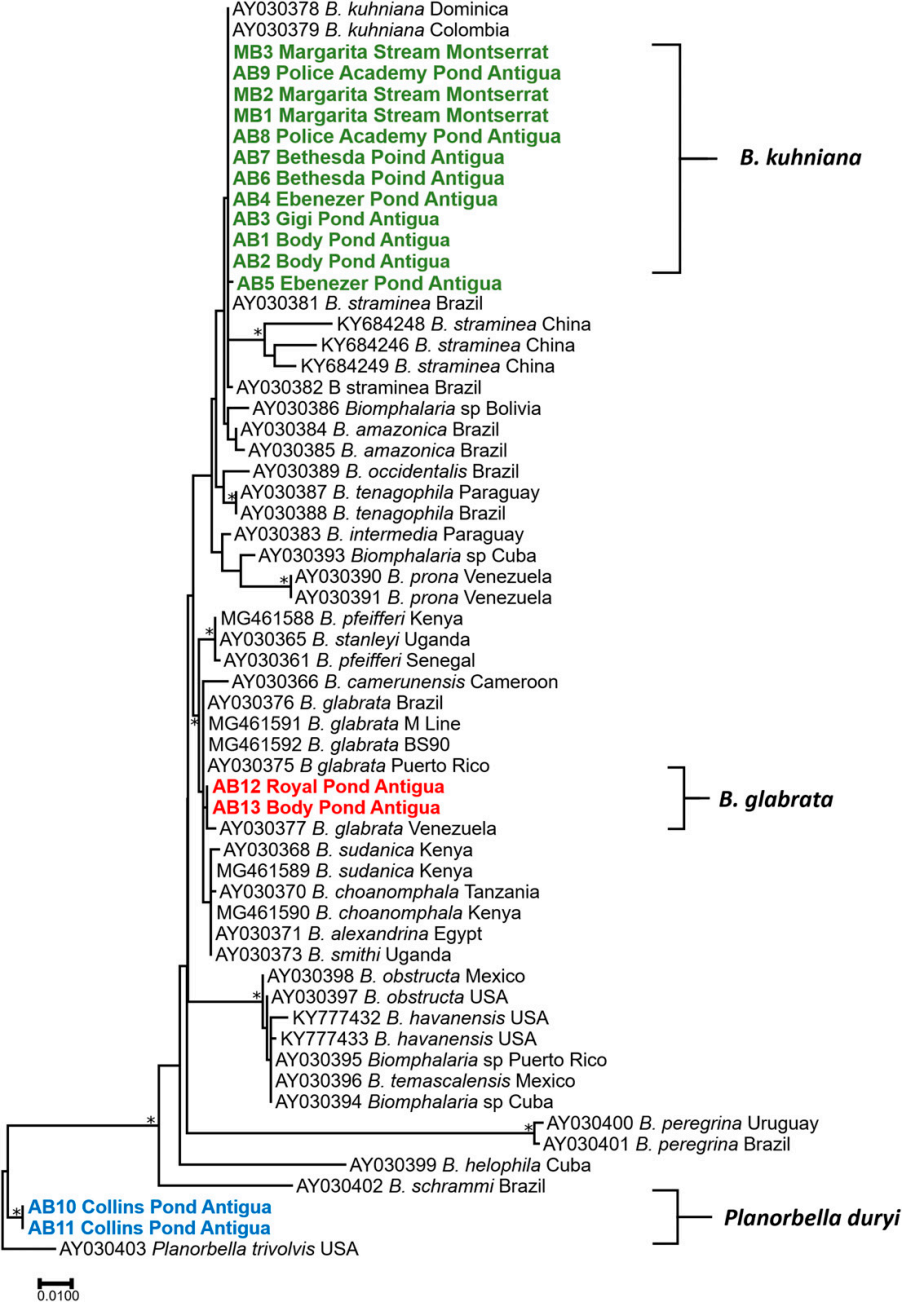
Phylogenetic tree based on 964 positions of partial 18S, ITS1, 5.8S, ITS2, and 28S genes. A total of 1,000 bootstraps were run, and model GTR + I + G was selected via model selection. * denotes nodes greater than 90%. Specimens obtained from Antigua and Montserrat are shown in bold and colored according to different species. ITSs = internal transcribed spacers. This figure appears in color at www.ajtmh.org.

Among the 15 localities sampled (Figure 3A), we collected 971 freshwater snails representing at least 12 different species, all of which were isolated and examined for trematode cercariae. All snail and cercariae samples are reported in Table 1. We found *Biomphalaria* (269) in seven localities. Identification of the snails was confirmed by morphological features, dissections, and by sequence data for ITSs (GenBank accession numbers MT753102–MT753117) and 16S marker genes (GenBank accession numbers MT753134–MT753149), which showed *Biomphalaria kuhniana* was present in six/seven localities and *B. glabrata* was present in two/seven localities (Figures 1 and 2). One snail conchologically resembling *B. glabrata* was collected from Royal Pond, and its identity was confirmed based on sequence data. A snail too small for specific morphological identification was collected from Body Pond and was confirmed by sequence data to be *B. glabrata*. All other *Biomphalaria* collected had shell anatomy and size, dissected genitalia including numbers of prostatic diverticuli, and sequence data for the two marker genes consistent with *B. kuhniana.*^6,21^ No *Biomphalaria* were found to be shedding schistosome cercariae, nor were they positive for *S. mansoni* as determined from the *ND*5 PCR assay. Two of 46 *B. kuhniana* from Body Pond were positive for digenetic trematode infections, one an echinostome and another a strigeid, and two of 107 *B. kuhniana* from Police Academy Pond were positive for strigeid infections. No other trematode infections were found among the Antiguan *Biomphalaria* snails we sampled. A dense population of *Planorbella duryi* was found in Collins Pond and is noteworthy because snails of this species can be readily confused with *B*. *glabrata* if dissections or sequence data are not used to differentiate them.

*Melanoides tuberculata* were collected from 6/15 sites on Antigua. None of the *M. tuberculata* were shedding trematodes. *Physa acuta* was collected from one locality, and *Physa marmorata* was collected at 10 different localities, and a few were positive for either strigeid or xiphidiocercaria infections. *Pseudosuccinea columella*, a known snail host for *Fasciola hepatica* in Cuba,^25^ was collected at four different localities, and none were shedding trematodes.

### Montserrat.

We sampled 14 different snail localities on Montserrat (Figure 3B) and from them collected 457 freshwater snails representing at least five different species, all of which were isolated and screened for trematode cercariae. We found 49 *Biomphalaria* in only one locality, Margarita stream. Sequence data for these snails confirmed their identification as *B. kuhniana* (Figures 1 and 2). Shell anatomy and size, and dissected genitalia including numbers of prostatic diverticuli were all consistent with *B. kuhniana*.^6^ None of the *B. kuhniana* released cercariae of any kind, including schistosomes. None were positive for *S. mansoni* using the *ND*5 PCR assay.

Of the 14 Montserrat locations sampled, *M. tuberculata* were found in eight localities, including Margarita Stream. *Physa marmorata* was also collected at 6/14 localities sampled and was the only snail species we found harboring trematode infections (echinostomes and strigeids) on the island. We did not collect any *P. columella* on Montserrat. No schistosome parasites of any kind were found among the snails we surveyed on either island.

## DISCUSSION

An important part of the process for verification of the elimination of human schistosomiasis is to gather data on the presence, distribution, and species composition of known or suspected schistosome vector snails and the schistosomes or other trematodes they may harbor. Although schistosomiasis elimination can be achieved even if appropriate snail vector species are still present, the status of vector populations is clearly germane to elimination and its prospects for long-term success. *Biomphalaria* snails were found in 7/15 localities sampled on Antigua and 1/14 localities sampled on Montserrat, most of which were *B. kuhniana*. To our knowledge, *B. kuhniana* has not been reported in the literature as a naturally infected vector of *S. mansoni* from any location.^26^ However, one article reported *Biomphalaria straminea* as naturally infected with *S. mansoni* in Venezuela,^27^ but these may have been *B. kuhniana.*^21^ Also, Paraense noted finding naturally infected *B. kuhniana* from Martinique (Pointier, personal communication), but this has not been verified. Experimental exposures of *B. kuhniana* to *S. mansoni* report a lack of compatibility.^28,29^

At the loci sequenced and analyzed, we found limited genetic variation between Antiguan and Montserratian *B. kuhniana* and Brazilian *B. straminea* (16S uncorrected *P*-distance value, *P* = 0.0515), the latter a known *S. mansoni* vector.^21,30,31^ Morphologically, there are also few well-defined features to distinguish these two taxa. Further experimental exposures are also clearly warranted because *B. kuhniana* has been reported widely from both the South American mainland and several islands of the Lesser Antilles,^6^ and its susceptibility to *S. mansoni* might vary with location or potentially be assisted by coinfections with other trematode species.

Although we did not find *Biomphalaria* infected with schistosomes, we found at least two different species of non-schistosome trematodes transmitted through *B. kuhniana* on Antigua. One is a strigeid of the genus *Apharyngostrigea* and the second an echinostome that closely resembles *Petasiger caribbensis* reported by Nassi^32^ from *B. glabrata* from Guadeloupe. Although *P. caribbensis* was first described from *B. glabrata*, it is shown here to be transmitted by *B. kuhniana*, which serves as a reminder that *S. mansoni* too could have a similar propensity to infect multiple *Biomphalaria* species.

We found two localities on Antigua that harbored *B. glabrata*, but we did not collect any *B*. *glabrata* on Montserrat, supporting reports^33^ that the eruption of the Soufrière Hills volcano eliminated its known habitats on the island.^33^ However, ours was a one-time collection trip, and we were only able to get to one site in the current exclusion zone, Bugby Hole, where *B. glabrata* was formerly found. Further sampling is recommended because *B*. *glabrata* occurs on Antigua only 54 km away, and introductions and extinctions are relatively common events on islands. People or birds may inadvertently transport this snail species to Montserrat as has happened with the exotic snail *M. tuberculata*, which has been known since at least 2001 on Montserrat.^17^

The widespread establishment of *M. tuberculata* on the islands (48% of localities surveyed) may preclude reintroductions of *B. glabrata* as it has been shown to be a potent competitor with *B*. *glabrata* on other Caribbean islands.^7,34^ Apart from one locality, Ebenezer’s Pond, we did not find *M. tuberculata* to coexist with *Biomphalaria* snails. *Physa marmorata* and *P. duryi* may also have the potential to displace *B. glabrata* or prevent its reestablishment because of their competitive ability.^35–37^ If populations of *B. glabrata* persist or are newly found on either island, then a difficult decision may need to be made by local public health and biodiversity experts. From the public health point of view, *B. glabrata* is an excellent vector for *S. mansoni*, but from a conservationist’s perspective, especially if *S. mansoni* is no longer present on the island, *B. glabrata* might be considered an endangered native species. Furthermore, if *B. glabrata* was deliberately rendered extinct on the islands, particular species of digenetic trematodes (other than *S. mansoni*) that depend on *B. glabrata* might suffer co-extinction, at least locally, unless they can infect other related species such as *B. kuhniana*. There are at least seven different species (excluding *S. mansoni*) of trematodes that are known to use *B. glabrata* as an intermediate host in the Caribbean region.^32,38,39^ Our comments should not be construed to mean elimination of snail vectors is a requirement for the elimination of schistosomiasis; indeed, preserving biodiversity like snails where possible should be encouraged.

Our survey prompted us to consider more broadly the needs of formal programs for declaration of elimination of schistosomiasis. In our view, such programs must include an accounting of the status of the snail side of the schistosome life cycle. It is tempting to conclude that if schistosome parasites are not found in the human population and are no longer a public health threat, then schistosomiasis is effectively eliminated. However, as the recent situation on the island of Corsica reminds us, schistosomes often have wild or domestic mammalian reservoir hosts that can serve as a source of infection for snails, leading to surprising reemergence of infections in humans.^18^ There is certainly a precedent for something similar to occur on the Caribbean islands as a focus of rodent-transmitted *S. mansoni* persisted for years in Guadeloupe after it ceased to be a human public health concern.^27,40^

As to whether vector snail species (or the schistosomes they may harbor) are actually present on the islands, this relates to a significant general problem for all elimination declarations. Such efforts are bedeviled by the fact that the hoped-for goal is to “find nothing,” raising the general question of “when is enough surveying and sampling enough?” This issue certainly applies to aspects of schistosomiasis occurring in the water. How then to proceed?

Resources for such endeavors will be limited, and sampling efforts will have to be adjusted depending on available budgets and the size of the country and number of localities requiring assessment. An important early step is to engage locals, educate them about the elimination verification process, and enlist their help in finding snail habitats. During our trip to Antigua and Montserrat, we were surprised about how many people knew of the purpose of our visit. On Montserrat, this was thanks to a brief interview session we held with the local radio station. Crowdsourcing initiatives involving cell phones and pictures of the relevant snails offer promise, but care is required as the process could be corrupted by expectation for rewards for finding the “right” snail species. Repeated visits to at least some and preferably all freshwater habitats are in order because the situation in them can change with season, recent rainfall, droughts, or modifications whether natural, like a volcanic eruption, or human mediated. Repeated visits may need to be guided by known hotspots of schistosome transmission identified from past studies. Certainly, habitats surrounding any villages known to have recently reported infected or seropositive persons or known to have proven populations of vectors like *B. glabrata* would be of interest. Studies like the recent serological survey in St. Lucia to detect lingering *S. mansoni* transmission can also provide valuable guidance for snail survey teams.^41^ If any snails were found to be infected with *S. mansoni*, then more concerted searching for other nearby pockets of infected snails and additional testing of local residents for evidence of infection would be warranted. It would also be useful to examine infected snails with respect to the timing of release of cercariae, as one indication of whether transmission is mediated by humans or possibly rodents.^42^ Release of cercariae at midday would be suggestive of a human-based transmission cycle, but biasing of cercarial release to the evening or early morning hours would be more suggestive transmission was being mediated by rodents. Even in the latter case, the potential for human infection would exist.

Sampling should include traditional snail isolation and shedding methods. With some training, this can ensure broad coverage of many snails. If possible, all collected snails should be held in aquaria and re-shed for cercariae after 2 weeks to determine if any snails may have had prepatent infections at the time of initial collection. This latter aspect has the disadvantage that snail culturing facilities may not be available, and some snails will die during the holding period. Alternatively, molecular xenomonitoring of snails for *S. mansoni* infection can be used,^24,43^ but some caution is required as this approach requires specialized equipment and reagents, requires validation for sensitivity and specificity of detection of *S. mansoni*, and may limit the number of snails that can be sampled, a disadvantage if snail populations are large. Pooling snails may help overcome these obstacles but are bound to reduce sensitivity of detection. Relative to elimination considerations, vector snail identifications should be based on both anatomical and molecular criteria. Such specimens have historical significance and should be deposited in museums, allowing ready access to the specimens for future reference and verification.

Availability and implementation of inexpensive alternative sampling strategies permitting wider or more frequent coverage would be a most welcome addition. Environmental DNA (eDNA) methods particularly offer promise for detecting the presence of either vector snails or schistosomes in water samples.^44–46^ Environmental DNA offers the advantages that signals in water samples are integrated across time and space permitting broader coverage, and specific identifications based on DNA sequences recovered can be acquired if desired. In our view, increased use of eDNA approaches is an important goal for future surveys. Most eDNA samples recovered will likely be negative, and well-chosen positive and negative controls will be critical to validate the process. Primer use will also need careful attention because other trematodes can co-occur and may result in false positives for *S. mansoni* without further verification via sequencing. Current eDNA procedures have not yet been validated across a broad range of transmission conditions, and because of the presumed rarity of *S. mansoni* in the Caribbean, this test environment will offer distinct challenges. Consequently, newer survey techniques are welcomed and encouraged but should be validated, in part by assessing their results alongside traditional specimen-based approaches involving collection, isolation, and shedding techniques followed by molecular identification as needed.

It is possible that repeated searches fail to recover any potential vector species, in which case the likelihood of indigenous transmission would be zero. If suspected vector species are found and their identifications confirmed, it would be helpful to know if they are compatible with local strains of *S. mansoni*. Such exposures need to be undertaken carefully under circumstances that preclude any possibility of *S. mansoni* reintroductions to the islands. Difficulties in obtaining necessary collection, exportation, and importation permits currently pose substantial hurdles to such endeavors, and assistance by the PAHO or WHO in helping secure such approvals would be very helpful.

Last, we must realize that nothing lasts forever, including declarations of elimination. Biological change is pervasive—introductions and reintroductions of snails including *B. glabrata* or other schistosome vectors, and of the schistosomes themselves, are always possible—especially on tropical islands. Local *Biomphalaria* species like *B. glabrata* that have been affected by introductions of competitor snails may be gone for good or might adapt and rebound. The backdrop of a rapidly changing climate is likely to have impacts we cannot presently foresee, which could favor or disfavor schistosomiasis transmission in novel ways. Nonetheless, efforts to certify elimination of schistosomiasis for the Caribbean nations are important because they can help us mark tangible progress in eliminating one of the most recalcitrant of all neglected tropical diseases and because they can be a testing ground for elimination declarations lying ahead in other nations.
